# Pre-Treatment Integrase Inhibitor Resistance and Natural Polymorphisms among HIV-1 Subtype C Infected Patients in Ethiopia

**DOI:** 10.3390/v14040729

**Published:** 2022-03-30

**Authors:** Dawit Assefa Arimide, Zsófia Ilona Szojka, Kidist Zealiyas, Atsbeha Gebreegziabxier, Fekadu Adugna, Sviataslau Sasinovich, Per Björkman, Patrik Medstrand

**Affiliations:** 1 Department of Translational Medicine, Lund University, 205 02 Malmo, Sweden; sviataslau.sasinovich@med.lu.se (S.S.); per.bjorkman@med.lu.se (P.B.); patrik.medstrand@med.lu.se (P.M.); 2 TB/HIV Department, Ethiopian Public Health Institute, Addis Ababa 1242, Ethiopia; kzealiyas@gmail.com (K.Z.); atsbehag@gmail.com (A.G.); 3 Department of Laboratory Medicine, Lund University, 222 42 Lund, Sweden; zsofia_ilona.szojka@med.lu.se; 4 NPO–HIV/AIDS, World Health Organization, Addis Ababa 3069, Ethiopia; adugnadadif@who.int; 5 Department of Infectious Diseases, Skane University Hospital, 205 02 Malmo, Sweden; 6 Clinical Microbiology and Infectious Disease Control, Skane University Hospital, 221 85 Lund, Sweden

**Keywords:** dolutegravir, integrase strand transfer inhibitor (INSTI), naturally occurring polymorphisms (NOPs), pretreatment, HIV drug resistance (HIVDR), docking, genetic barrier, Ethiopia

## Abstract

Dolutegravir-based antiretroviral therapy (ART) has been scaled up in many developing countries, including Ethiopia. However, subtype-dependent polymorphic differences might influence the occurrence of HIV-drug-resistance mutations (HIVDRMs). We analyzed the prevalence of pre-treatment integrase strand transfer inhibitor (INSTI) HIVDRMs and naturally occurring polymorphisms (NOPs) of the integrase gene, using plasma samples collected as part of the national HIVDR survey in Ethiopia in 2017. We included a total of 460 HIV-1 integrase gene sequences from INSTI-naïve (*n* = 373 ART-naïve and *n* = 87 ART-experienced) patients. No dolutegravir-associated HIVDRMs were detected, regardless of previous exposure to ART. However, we found E92G in one ART-naïve patient specimen and accessory mutations in 20/460 (4.3%) of the specimens. Moreover, among the 288 integrase amino acid positions of the subtype C, 187/288 (64.9%) were conserved (<1.0% variability). Analysis of the genetic barrier showed that the Q148H/K/R dolutegravir resistance pathway was less selected in subtype C. Docking analysis of the dolutegravir showed that protease- and reverse-transcriptase-associated HIVDRMs did not affect the native structure of the HIV-1 integrase. Our results support the implementation of a wide scale-up of dolutegravir-based regimes. However, the detection of polymorphisms contributing to INSTI warrants the continuous surveillance of INSTI resistance.

## 1. Introduction

Following the global increase of pre-treatment drug resistance (PDR) to non-nucleoside reverse transcriptase inhibitors (NNRTIs), the World Health Organization (WHO) recommended the transition from NNRTI to integrase strand transfer inhibitor (INSTI)-based regimens in both treatment-naïve and treatment-experienced patients [1,2,3]. Several low- and middle-income countries have already transitioned to the dolutegravir (DTG)-based regimen, and many more are in the planning phase, so millions of people living with HIV will soon receive DTG combined with two nucleoside reverse transcriptase inhibitors (NRTIs) as first- and second-line therapies [2,4].

HIV-1 integrase (IN), which comprises 288 amino acids encoded by the 5′-end of the HIV *pol* (polymerase) gene, plays a vital role in HIV-1 replication by catalyzing two distinct reactions: 3′-end processing and strand transfer [5,6,7]. IN consists of three functional domains: the N-terminal domain (NTD) (aa: 1–50), which contains a highly conserved histidine–histidine–cysteine–cysteine (H_12_H_16_C_40_C_43_) motif that coordinates zinc binding and favors multimerization of the IN subunit [8]; the catalytic core domain (CCD) (aa: 51–212), which contains the catalytic triad D_64_D_11_6E_152_ (known as the DDE motif) that plays an essential role in IN enzymatic activity; and the C-terminal domain (CTD) (aa: 213–288), which is involved in binding to viral and cellular DNA, and in protein oligomerization and interactions with the reverse transcriptase [5,6,7,9].

INSTIs inhibit the HIV-1 integrase strand transfer steps to block the integration of HIV viral DNA into the host cell chromosomal DNA through competitive binding to the enzyme’s active site [7,10]. There are currently five US Food and Drug Administration (FDA)-approved drugs belonging to this therapeutic class: raltegravir (RAL), elvitegravir (EVG), DTG, bictegravir (BIC), and cabotegravir (CAB) [11]. RAL and EVG were the first-generation INSTIs to be used clinically; however, their relatively low genetic barrier for resistance and the extensive cross-resistance between them limit their efficiency [12,13]. DTG and BIC are second-generation INSTIs shown to be highly effective in both treatment-naive and treatment-experienced individuals with good tolerability and a high genetic barrier to resistance [12,14]. Pooled analysis of resistance data conducted by Yang et al. (2019) indicated that the development of resistance to DTG and BIC was rare [12]. However, with the wide scale-up of DTG, gradual development and transmission of HIVDR against INSTIs will be inevitable and can render existing therapies ineffective, thereby increasing the risk of virological failure, disease progression, and mortality [12,15,16,17,18,19,20,21,22,23,24,25,26,27,28].

Although non-B subtypes dominate the global HIV epidemic, most clinical and virological studies on DTG were based on subtype B. However, subtype-dependent differences in naturally occurring polymorphisms (NOPs) have been implicated in the development of different mutational pathways, leading to varying levels of drug resistance against INSTIs among different HIV-1 subtypes [5,13,29,30,31,32,33,34]. Q148H and G140S, which confer resistance to RAL and EVG and cross-resistance to DTG, appear more frequently in subtype B than in non-B subtypes [31]. Similarly, R263K is mainly present in subtype B, while G118R has a pathway in selecting DTG resistance in non-B subtype viruses [13,22,35,36]. 

HIV-1 sequences and structure-based analyses also showed that subtype-specific NOPs, especially at the active site of IN, can affect the genetic barrier to drug resistance by influencing the selection of resistance mutations, native protein structure, and the function of the drug-mediated inhibition of the enzyme [29,30,32,33,37]. 

In 2019, an estimated 669,236 people were living with HIV in Ethiopia, and the epidemic was dominated by subtype C [38,39]. Similar to many other countries in sub-Saharan Africa, Ethiopia has implemented the test-and-treat strategy, with DTG-based regimens recommended as the first-line antiretroviral therapy (ART) [40]. However, there is limited knowledge of the frequency and characteristics of NOPs of IN or their effect on the development of INSTI resistance. This study aimed to investigate HIV-1 IN genotypic profile to evaluate the prevalence of pre-treatment DRMs and NOPs that might affect the genetic barrier to the emergence of resistance in INTSI-naïve patients in Ethiopia infected with HIV-1 subtype C.

## 2. Materials and Methods

### 2.1. Study Design

In this study, we used plasma samples collected from HIV-1-infected patients as part of a national HIVDR survey conducted in Ethiopia. A cross-sectional survey was conducted in 2017 among treatment-naïve patients and patients on first- and second-line regimens in selected health facilities from different parts of the country according to the WHO-recommended HIVDR survey [41]. After obtaining written informed consent from each participant, 10 mL of blood was collected by venipuncture for CD4+ T-cell count, viral load, and HIVDR genotyping. Basic demographic and clinical information were also collected during the survey using a standardized questionnaire. Specimens were transported to the Ethiopian Public Health Institute (EPHI) on dry ice for viral load testing and long-term storage at −80 °C. HIV-1 VL was determined using the Abbott RealTime HIV-1 assay (Abbott Molecular Inc., Des Plaines, IL, USA). Using 1000 copies/mL as a viral load suppression threshold based on the WHO recommendation [42], all samples with a viral load ≥1000 copies/mL were then shipped to the National Institute of Respiratory Diseases-Mexico (INER) laboratory for HIVDR genotyping.

### 2.2. HIV-1 Genotyping

Genotyping of the integrase region was performed using an in-house-developed and -validated protocol for IN [43]. Amplicons obtained by the nested PCR method were used for Sanger sequencing using the BigDye technology on the ABI Prism 3730 Genetic Analyzer (Applied Biosystems, Foster City, CA, USA). Sequence assembly and editing were performed using the RECall V 2.0 HIV-1 sequencing analysis tool (University of British Columbia, Vancouver, BC, Canada) [44]. Sequence quality control was performed using the WHO tool (https://sequenceqc-dev.bccfe.ca/who_qc (accessed on 28 June 2021)) and the Quality Control program of the Los Alamos HIV sequence database (https://www.hiv.lanl.gov (accessed on 28 June 2021)).

### 2.3. Subtype Determination Using HIV-1 Integrase Sequences

The HIV-1 subtyping was performed using the online automated subtyping tools REGA v3.0 [45], COMET [46], and the jumping profile Hidden Markov Model (jpHMM) [47]. Subtyping was further confirmed by Maximum likelihood (ML) phylogenetic tree analysis with the IN references sequences from HIV-1 subtype (A-K) and recombinant virus downloaded from the Los Alamos database (http://www.hiv.lanl.gov (accessed on 3 July 2021)). Multiple sequence alignment was conducted using MAFFT version 7 [48] and was then manually edited using BioEdit V7.0.9.0 [49,50] until a perfect codon alignment was obtained. ML tree topology was constructed using the online version of PhyML v 3.0 [51] with the GTR+I+Γ nucleotide-substitution model (using the estimated proportion of invariable sites and four gamma categories). A heuristic tree search was performed using the SPR branch-swapping algorithm. Branch support was determined with aLRT-SH (approximate likelihood ratio test, Shimodaira Hasegawa-like) [52]. Clusters were defined as monophyletic clades with aLRT-SH support ≥0.9. The subtype-resolved ML phylogeny trees were visualized using the FigTree v1.4.0 program. Sequence(s) that formed a cluster with the reference sequences belonging to the same subtype were assigned to that subtype.

### 2.4. HIV-1 Drug Resistance Analysis

INSTI-associated mutations were identified using the Stanford HIV Drug Resistance Database (HIVdB v9.0) (https://hivdb.stanford.edu/hivdb/by-mutations (accessed on 7 July 2021)). INSTI DRMs were categorized as major resistance mutations, accessory resistance mutations, and other mutations according to the Stanford HIV Drug Resistance Database. Major resistance mutations were primarily nonpolymorphic DRMs that caused a significant reduction in INSTI susceptibility, even when they occurred alone. Accessory mutations were nonpolymorphic or minimally polymorphic mutations that caused only low-level reduction of INSTI susceptibility when they occurred alone, but may have augmented resistance and/or restored the fitness of viral mutants with major resistance mutations. The other mutations included highly polymorphic and/or rare nonpolymorphic mutations that may have been weakly associated (uncertain role) with drug resistance. We further extensively investigated all amino acid positions associated with decreased INSTI susceptibility. Samples harboring resistant and/or a mixture of wild-type and resistant amino acids were considered resistant.

### 2.5. HIV-1 Subtype C Integrase Polymorphism and Conservation Analysis

For this analysis, only HIV-1 subtype C sequences were used. Briefly, multiple sequence alignment was conducted using MAFFT version 7 [48] and was then manually edited using BioEdit V7.0.9.0 [49,50] until a perfect codon alignment was obtained. The nucleotide sequences were translated to an amino acid sequence. Then, each amino acid along the 288 IN positions was extensively investigated for the presence of primary mutations and of nonpolymorphic and polymorphic mutations associated with resistance to INSTI. The prevalence of each amino acid at each IN position was determined and compared to the HIV-1 subtype B reference sequence (GenBank accession number: K03455). We defined NOPs as substitutions within the HIV-1 IN that occurred in ≥1% of the sequences for this analysis [6]. The positions with ≥20% substitutions were defined as highly polymorphic, while those with ≤0.5% variability were considered highly conserved.

### 2.6. Generation of Consensus HIV-1 Integrase Sequence

To comprehensively describe the variability (polymorphism) in the IN sequences, we downloaded global subtype B and C IN sequences that matched the region (HXB2: 4230- 5093 relative to HXB2 clone) from the HIV Los Alamos National Library (LANL) database (https://www.hiv.lanl.gov (accessed on 13 July 2021)). To avoid the overestimation of variant calling and ensure the sequences included in the analysis were from INSTI-naïve patients, only sequences before 2007 (before the FDA approved INSTIs) were used. The quality of all HIV-1 sequences was verified using the online Quality Control program (http://www.hiv.lanl.gov (accessed on 14 July 2021)). Sequences with stop codons and/or frameshifts and/or poor quality were removed from the analysis. Only one sequence per patient was retained. For a patient with multiple sequences, the earliest sequence was selected and used. The consensus amino acid sequence for IN was generated for Ethiopian HIV-1 subtype C, the global HIV-1 subtype B, and the global subtype C sequence using BioEdit V7.0.9.0 [49,50]. For positions where two amino acids occurred at frequencies higher than 30%, both amino acids were represented, and the first letter seen at the consensus represented the most prevalent amino acid. 

Furthermore, to assess the impact of previous exposure to ART on IN gene NOPs, the consensus amino acid sequences of IN from the ART-naïve and ART-experienced patients were generated and compared. Similarly, we also compared the consensus amino acid sequences of IN from patients with one or more major HIVDRMs to protease inhibitor (PI), NRTI, and/or NNRTIs (HIVDR group) with those with no major HIVDRMs (no-HIVDR groups) in their corresponding protease/reverse transcriptase (PR/RT) gene. 

### 2.7. Genetic Barrier to Integrase Strand-Transfer Inhibitor Resistance

To assess differences in the genetic barrier for evolution of drug-resistance substitutions between subtypes C and subtype B, we compared Ethiopian HIV-1 subtype C IN sequences obtained from INSTI-naïve patients and global HIV-1 subtype B sequences obtained from LANL (INSTI-naïve, collected before 2007). We calculated the genetic barrier to INSTI resistance for 10 major INSTI resistance amino acid positions (19 substitutions) using a previously published method [53]. Briefly, we first determined the extent of natural diversity at each selected position in our dataset of Ethiopian HIV-1 subtype C IN sequences and global subtype B IN sequences by identifying all wild-type triplets and their prevalence. Next, we compute genetic barrier score for each wild-type triplet to evolve to resistant amino acid at the specific selected position. The genetic barrier was calculated as the sum of transitions and/or transversions required to evolve to any major drug-resistance substitution. We used a score of 1 for transition (A↔G and C↔T), 2.5 for transversion (A↔C, A↔T, G↔C, G↔T), and 0 when no change was needed, as described by Nguyen et al. (2012) [53]. The smallest number (minimal score) of transversion and/or transition required for evolution from wild-type codon to resistant codon were used to calculate the genetic barrier. 

### 2.8. Modeling and In Silico Predictions of HIV-1 Integrase and Dolutegravir Interaction

For in silico predictions, 20 randomly selected (10 from each ART-naïve (PDR) and ART-experienced (ADR)) sequences were used. The ART-naïve IN sequences used in our analysis had no HIVDRMs against NRTI, NNRTI, and/or PI in their corresponding PR/RT gene, while the ART-experienced group had one or more HIVDRMs against NRTI, NNRTI, and/or PI. A multiple-sequence alignment of amino acid sequences (without any gap) was made using ClustalW (https://www.genome.jp/tools-bin/clustalw (accessed on 1 November 2021)). An amino acid identity matrix was created with Clustal 12.1 (https://www.ebi.ac.uk/Tools/msa/clustalo (accessed on 1 November 2021)) and visualized using GraphPad Prism 8. 

The crystallographic structure of full-length HIV-1 IN (accession number: 6u8q.pdb) was obtained from the Protein Data Bank (www.rcsb.org (accessed on 2 November 2021)) [54]. To visualize both the PDR and ADR HIV-1 IN, the 6u8q was modified by using UCSF-Chimera at 12 amino acid positions (see Table S2), and a monomer was used in the docking prediction. The structure (6u8q) originally included a DNA fragment and DTG. After removing all ligands, the DNA fragment and water molecules from the crystal structure, receptor, and ligand–DTG files were separately saved for further analysis. MGL Tools (Version 1.5.7rc1) was used for creating .pdbqt files of the receptor and ligands needed for docking with Autodock Vina (Vina) (Version 1.1.2) [55,56]. Ligands were docked to the binding site cavity using x = 211, 63 Å; y = 205, 453 Å; and z = 171, 895 Å Cartesian coordinates that used the catalytic site in the monomer of HIV-1 IN. The grid box dimensions used for the search space were 50 Å × 40 Å × 40 Å. Docking calculations were performed with an exhaustiveness option of 8 (average accuracy) and an energy range of 3. Validation of the docking method was performed by redocking DTG to the modified crystal structure to the modified above-mentioned structure.

### 2.9. Statistical Analysis

Fisher’s exact test, the Chi-squared test, and the Mann–Whitney U-test were used to evaluate the statistical differences between groups. *p*-values ≤ 0.05 were considered statistically significant. 

## 3. Results

A total of 460 IN sequences obtained from INSTI-naïve patients were included in the analysis. Among these, 373 sequences were from patients who did not report exposure to any antiretroviral drug at the time of specimen collection (ART-naive), while 87 sequences were from ART-experienced (NNRT-based or PI-based regimens) patients, with virological failure (viral load ≥ 1000 copies/mL) while on a first-line (*n* = 41) or second-line (*n* = 46) regimen. 

### 3.1. HIV-1 Subtyping

**Figure 1:**
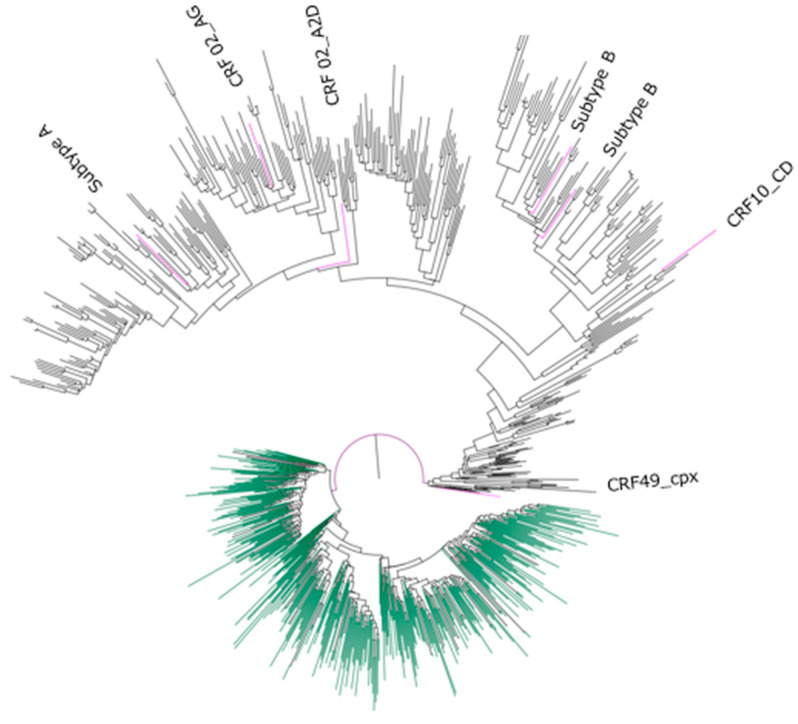
Maximum-likelihood phylogenetic tree of HIV-1 viral strains circulating in Ethiopia using integrase (IN) sequences.

Online subtyping and the subsequent phylogenetic analysis results showed that 98.5% (453/460) of the sequences were subtype C, while 0.43% (2/460), 0.22% (1/460), 0.22% (1/460), 0.22% (1/460), 0.22% (1/460), and 0.22% (1/460) were subtype B, subtype A1, CRF10_CD, CRF02_AG, CRF49_cpx and CRF_A2D, respectively (Figure 1).

The phylogenetic tree in Figure 1 contains a total of 874 sequences, including Ethiopian sequences (*n* = 460) and (*n* = 414) integrase reference sequences for HIV-1 subtypes (A–K) and circulating recombinant forms downloaded from the HIV-1 LANL database. An ML tree was constructed using the online version of PhyML v 3.0. The reference sequences from the Los Alamos National Laboratory are in black in the figure. All the Ethiopian sequence’s clusters with the HIV-1 subtype C reference sequence are in green, while the non-subtype C Ethiopian sequences are in pink.

### 3.2. Prevalence of Major Integrase Strand-Transfer Inhibitor Resistance Mutations

No major DRMs known to be associated with DTG resistance (T66K, E92Q, G118R, E138K/A/T, G140S/A/C, Q148H/R/K, N155H, or R263K) were detected among INSTI- naïve individuals, regardless of previous exposure to ART. However, one (0.22%) sequence from a person without previous ART exposure was found to harbor E92G, a mutation that moderately reduces EVG susceptibility but does not reduce susceptibility to RAL and DTG. 

A total of 4.4% (20/460) of the sequences contained five different IN accessory mutations: −E157Q (2.39%), G163R/K (0.65%), Q95K (0.65%), T97A (0.43%), and G149A (0.22%). There was no significant difference in the prevalence of accessory mutations among ART-naïve and ART-experienced patients (*p* = 0.9) (Table 1). Only one accessory mutation per sequence was detected, except for one sequence with two (G149A and E157Q) accessory mutations. In addition, other mutations including M50I (18.5%, 85/460), L74I/M (2.8%, 13/460), S119R, (0.9%,4/460), V151I, (1.3%, 6/460), and D230N (0.4%, 2/460) were also detected.

### 3.3. Integrase Strand-Transfer Inhibitor Resistance among Patents on Antiretroviral Therapy

**Figure 2:**
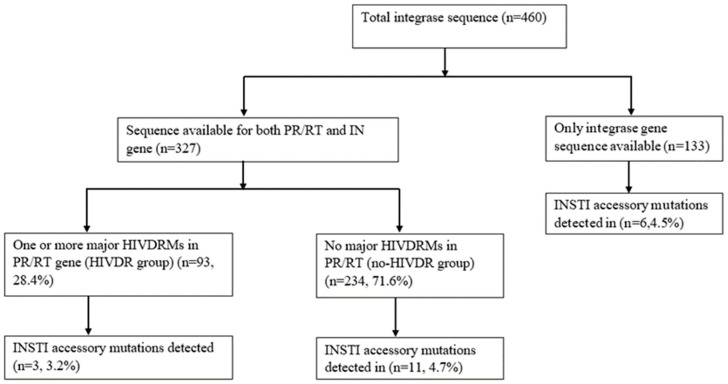
Flow chart of study-participant selection and INSTI drug resistance among INSTI-naïve patients (*n* = 460). Abbreviations: PR/RT, protease and reverse transcriptase gene; IN, integrase; INSTI, integrase strand-transfer inhibitors; HIVDRM, HIV-drug-resistance mutations.

To assess the impact of ART exposure to NRTI, NNRTI, and/or PI on the selection of INSTI-resistance mutations, we further compared the INSTI HIVDRMs from patients with one or more major HIVDR mutations to NRTI, NNRTI, and/or PI (HIVDR group) with those with no HIVDRMs in their corresponding PR/RT genes (no-HIVDR group) (Figure 2).

Briefly, among the total 460 IN sequences used in our analysis, 327 had a corresponding PR/RT gene sequence, of which 234 had no major HIVDRMs (no-HIVDR group), while 93 of the sequences (HIVDR group) had one or more HIVDRMs against the NRTI, NNRTI, and/or PI (see Table S1). No major INSTI HIVDRMs were detected in either of these groups, and there was no significant difference in the presence of accessory mutations with regard to previous ART exposure, nor with regard to DRMs toward other ARVs. Among the HIVDR and no-HIVDR groups, 3.2% (3/93) and 4.7% (11/234) accessory mutations were detected, respectively (*p* = 0.8); while 4.29% (15/373) and 5.75% (5/87) accessory mutations were detected among ART-naïve and ART-experienced groups, respectively (*p* = 0.6).

**Figure 3:**
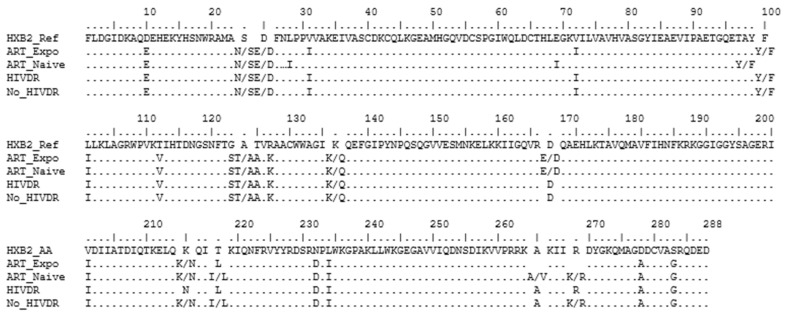
Alignment of Ethiopian HIV-1 subtype C integrase (IN) consensus sequence. The consensus sequence from the ART-naïve sequences (*n* = 367) is represented as ART_Naive, and that from ART-experienced (*n* = 87) is represented as ART_Expo. The consensus sequence from the sequence with no HIVDR mutation in the protease/reverse transcriptase (PR/RT) gene (*n* = 234) is represented as No_HIVDR, while that with one or more major mutation in PR/RT is represented as HIVDR. Positions with more than one amino acid are both represented. HXB2 represents the consensus HIV-1 subtype B reference sequence from the LANL database (accession number: K03455).

High similarity was also observed when comparing the consensus sequence from ART-naïve and ART-experienced patients, as shown in Figure 3. Similarly, our comparison of the consensus sequences from the HIVDR and non-HIVDR groups also showed high similarity between the two consensus sequences, except at positions K215N, T218L, and R269, where the HIVDR group had one amino acid; while the no-HIVDR group had a mixture of amino acids at positions T215K/N, T218I/L, and R269R/K, respectively (Figure 3).

### 3.4. Prevalence of Naturally Occurring Integrase Polymorphisms in HIV-1 Subtype C

**Figure 4:**
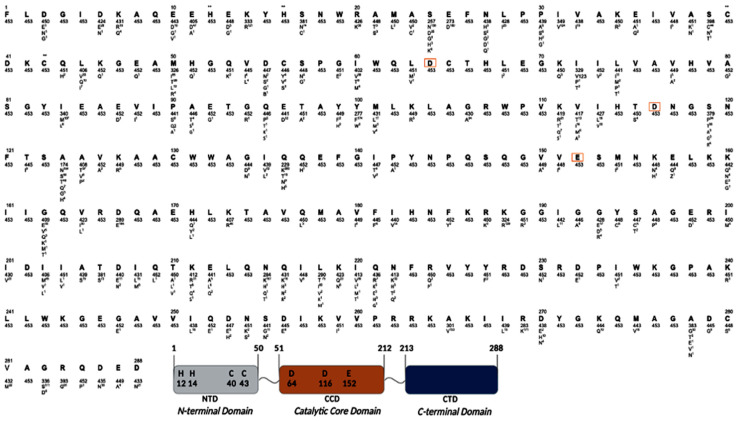
Distribution of variants among HIV-1 subtype C integrase sequences. The number at the top is the amino acid position in the integrase gene (1–288), and the consensus subtype B sequence is indicated below the number. Beneath the consensus, the number indicates the number of sequences containing the amino acid at the indicated position. The variant amino acid at each position is indicated along with number of sequences with that amino acid (superscript). The HHCC zinc-binding motifs are indicated by **, the amino acid of the DDE active sites are indicated by red boxes. The N-terminal domain is indicated in gray at positions 1–50, while the catalytic core domain (CCD) at positions 51–212 is indicated in orange, and the C-terminal domain (CTD) at positions 213–288 is indicated in blue.

An alignment of the 453 HIV-1 subtype C IN sequences from the INSTI-naïve Ethiopian patients was extensively analyzed and compared to the HIV-1 subtype B reference sequence (GenBank accession number: K03455). Based on our definition of polymorphism (≥1.0% variability), an overall 64.9% (187/288) amino acid positions of the IN were conserved. The conservations of the NTD, CCD, and CTD were 60% (30/60), 66.1% (107/162), and 66.8% (50/76), respectively. The distribution of polymorphisms in 453 HIV-1 subtype C IN sequences is shown in Figure 4.

### 3.5. Analysis of the N-Terminal Domain (NTD)

Within the NTD, the Zn-binding motif (H_12_H_16_C_40_C_43_) involved in the multimerization of the IN subunit, stabilization of folding, and interaction with LEDGF/p75 were highly conserved [6]. However, amino acid positions, D10E, S24N, D25E, V31I, and M50I were highly polymorphic (>20.0% variability). We also observed that the residue E10 had been replaced by D (aspartic acid) in 97.8% of sequences, which might be the signature of subtype C (Figure 4).

### 3.6. Analysis of the Catalytic Core Domain (CCD)

In the CCD, the catalytic triad D_64_D_116_E_152_ was highly conserved, and was found within the conserved regions 61–70, 114–118, and 152–155, respectively. The critical positions for DNA-binding HIV-1 integration and replication (Q62, H67, N120, N144, Q148, and N155) [57] and the residue involved in the chemical bond and hydrophobic contact with the LEDGF/p75 [6] (A128-A129-W131-W132-Q168-E170-T174-M178) were also highly conserved. However, amino acids at codon positions G163, V165, D167, H171, and K173 within the I161-K173 region known to be involved in the noncanonical nuclear localization signal [6,22], and the K188 within the KRK motif (K_186_, R_187_, K_188_), which is vital for the integrase:integrase:oligomerization at the dimer:dimer interface [6,22], showed 28.5% variability. 

Among the INSTI-mutation positions in the CCD residues that directly reduced the INTSI susceptibility, H51, T66, E92, F121, G140, Y143, Q146, S147, Q148, S153, N155, and E157Q were highly conserved, except for codon position E157Q, which was a polymorphic position (>1.0% variability). However, a highly polymorphic residue in the CCD including V72I, I84M, F100Y, L101I, T112V, T124A, T125A, R127K, K136Q, D167E, K188R, and V201I was observed. 

### 3.7. Analysis of the C-Terminal Domain (CTD)

Within CTD, the two large consecutive residues, L241-Q252 and I257-K264, which are involved in the binding of viral and cellular DNA, were found to be highly conserved, except for positions I251 and V257, which were mutated to I251L and V259I in 3.5% and 0.7% of the sequences, respectively. However, the important positions for DNA binding and integrase multimerization (K258, V260, R262, R263, and K264) [6] were fully conserved.

Our analysis also showed that 24 amino acid positions were highly polymorphic (>20.0% variability): D10E, K14R, S24N, D25E, V31I, M50I, V72I, I84M, F100Y, L101I, T112V, T124A, T125A, R127K, K136Q, D167E, K188R, V201I, K215N, T218I, A265V, R269K, D278A, and S283G. Six of these (D10E, K14R, S24N, D25E, V31I, and M50I) belonged to the NTD, whereas 12 (V72I, I84M, F100Y, L101I, T112V, T124A, T125A, R127K, K136Q, D167E, K188R, and V201I) belonged to the CCD, and the other 6 (K215N, T218I, A265V, R269K, D278A, and S283G) belonged to the CTD. 

**Figure 5:**
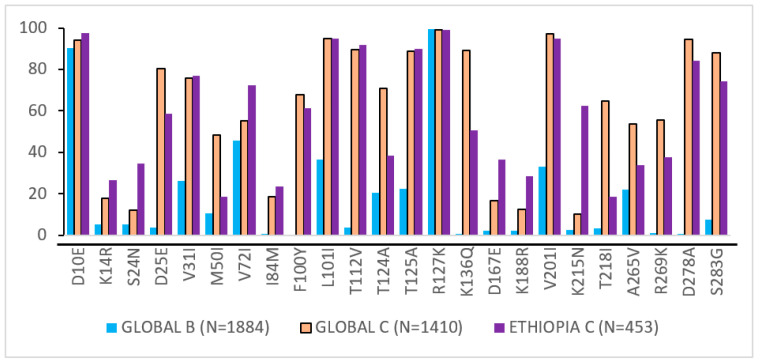
Prevalence of integrase highly polymorphic mutation in INSTI-naïve patients. The comparison was done using sequences downloaded from the Los Alamos National Library (LANL) database for subtype B (*n* = 1884), HIV-1 subtype C (*n* = 1410), and Ethiopian subtype C (*n* = 453).

Our comparison of the NOPs’ distribution with the global subtype B and global subtype C sequences downloaded from LANL showed that the Ethiopian HIV-1 subtype C IN sequences had a high similarity to the global subtype C sequence, but were quite different from the global subtype B, as shown in Figure 5.

### 3.8. Analysis of the Subtype Consensus Integrase Sequences

**Figure 6:**
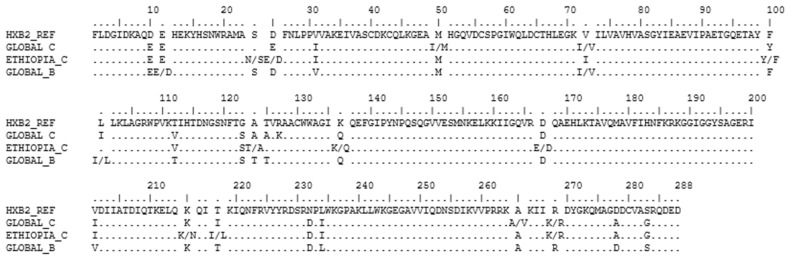
Alignment of integrase (IN) consensus sequence from LANL database (https://www.hiv.lanl.gov (accessed on 25 July 2021)). The consensus sequence from the global HIV-1 subtype C (*n* = 1410 sequences) is represented as GLOBAL C, the global HIV-1 subtype B (*n* = 1884 sequences) is represented as GLOBAL_B, and the Ethiopian subtype C sequence (*n* = 453 sequences) is represented as ETHIOPIA_C. Positions with more than one amino acid are both represented. HXB2 represents the consensus HIV-1 subtype B reference sequence from the LANL database (accession number: K03455).

The consensus IN sequence for the global HIV-1 subtype B and global subtype C were generated using 1884 and 1410 sequences, respectively. Our comparison of the 288 amino acid sequence alignment of the consensus Ethiopian HIV-1 subtype C with the global HIV-1 subtype C showed high similarity, except for the mixture of amino acid sequences at positions 25E/D, 100Y/F,124T/A, 136K/Q, 167E/D, 215K/N, and 218I/L in the Ethiopian consensus; and 50M/I, 72I/V, and 265A/V in the global HIV-1 subtype C consensus sequence. However, it differed from the global subtype B consensus at eight positions with complete amino acid replacement (31, 112, 125, 201, 218, 234, 278, 283), while a mixture of amino acids was detected at positions 11E/D, 72I/V, and 101I/L in the global subtype B consensus sequences and at 24N/S, 25E/D, 100Y/F, 124T/A, 136K/Q, 167E/D, 215K/N, and 269K/R in the Ethiopian subtype C consensus sequence (Figure 6).

### 3.9. Genetic Barrier to Dolutegravir Resistance

In this study,19 substitutions conferring major resistance to DTG at 10 amino acid positions in the IN (T66A/I/K, E92G, G118R, E138K/A/T, G140S/A/C, Y143R/C/H, S147G, Q148H/R/K, N155H, and R263K) were assessed to explore the genetic barrier to DTG. For each codon, the number of transitions and/or transversions required for a IN drug resistance associated substitution were calculated. A total of 1884 global HIV-1 subtype B sequences and 453 Ethiopian subtype C sequences from INSTI-naïve patients were compared for differences in the genetic barrier to INSTI resistance (Table 2).

Overall, the sequence analysis of the two subtypes showed similar predominant codon use at the selected amino acid positions, resulting in a similar minimum score for the genetic barrier to DTG. However, at position 140, the predominant codons in subtype C were GGG (53.6%) and GGA (45.9%). In contrast, in subtype B, GGC (85.0%) was the predominant codon resulting in a difference in the calculated genetic barrier at this position. For subtype C, two transversions (minimum score of 5) were required to mutate to G140C (GGG/A to ATG/C); while for subtype B, one transversion and transition (minimum score: 3.5) were required to mutate to G140C (GGC to TGT). Similarly, a two-point mutation (one transversion and one transition) (minimum score of 3.5) was required to mutate to G140S (GGG/A to AGT/C) for subtype C; while subtype B required a one-step transition (minimum score of 1) (GGC to AGC). 

### 3.10. Impact of Protease and Reverse-Transcriptase Drug-Resistance Mutation on the Structure of HIV-1 Integrase

**Figure 7:**
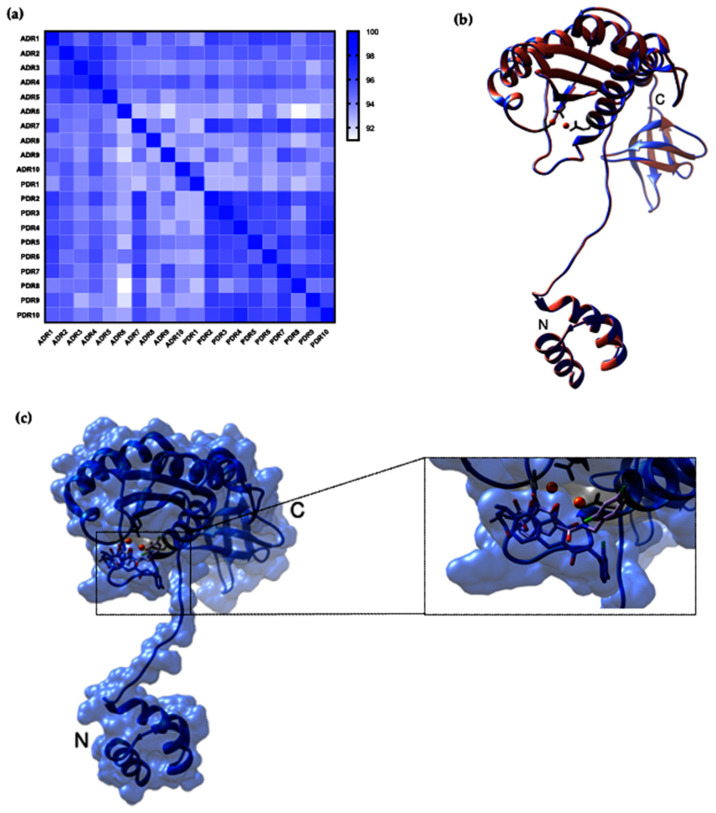
Visualization of HIV-1 IN amino acid sequences from ART-naive (PDR) and ART-experienced drug-resistant (ADR) individuals. (**a**) Heat map of amino acid identities of HIV-1 IN amino acid sequences from PDR and ADR individuals. The heat map was generated using a percent identity matrix table created in Clustal 12.1, and the heat map was visualized in GraphPad Prism 8. (**b**) For in silico predictions, a molecular model of the monomeric HIV-1 integrase structure 6u8q was used and modified based on the multiple-sequence alignment of amino acid sequences of 10 ADR and 10 PDR sequences. Figures were created in Chimera (https://www.cgl.ucsf.edu/chimera https://www.cgl.ucsf.edu/chimera (accessed on 2 November 2021)). PDR (salmon) and ADR (cornflower blue) HIV-1 monomers are represented as ribbons, the catalytic triad is represented with sticks in light grey, and magnesium is represented in orange-red. The PDR structure was moved at 0.01 Å on the x-axis. (**c**) Surface views of the structure and the validation of docking were conducted using Autodock Vina. Dolutegravir is represented with sticks; blue shows the original coordinates, and purple shows the docking mode of dolutegravir in Autodock Vina.

The effects of HIVDRMs in HIV-1 PR and/or RT on the secondary structure of HIV-1 IN were investigated on 20 sequences: 10 from ART-naïve (PDR) and 10 from ART-experienced (ADR) individuals representative of randomly selected HIV-1 IN sequences. The sequence identity matrix (Figure 7a) showed that all the sequences were more than 92% identical at the amino acid level, and there were no major differences between the two main groups. To study the effects of PR and RT drug-induced resistance on the structure of HIV-1 IN, chain A of the 6u8q structure was modified at 12 positions to represent both the ADR and the PDR sequences (see Table S2). The alignment of the monomers of the PDR and ADR INs did not result in any differences between the two groups. DTG was successfully docked to both the PDR and ADR IN by Autodock Vina (Figure 7c), and the docking score was −6.5 kcal/mol, which was at a similar position as the original DTG ligand.

## 4. Discussion

Overall, our results revealed no major DTG associated HIVDRM mutations among INSTI-naïve individuals, regardless of previous exposure to ART. In one individual, the E92EG mutation was found, which moderately reduced EVG susceptibility, but had no effect on DTG. However, INSTI accessory mutations and NOPs, which could influence INSTI susceptibility and the genetic barrier to INSTI resistance, were detected. Our polymorphism analysis showed that 64.9% (187/288) of amino acid positions of the HIV-1 subtype C IN sequences from INSTI-naïve individuals were conserved (<1.0% variability). The majority of amino acids involved in key functions of the enzyme (the HHCC motif and the DDE motifs [6,22]) were fully conserved. The genetic barriers to DTG resistance were similar at selected amino acid positions for subtypes B and C, except that subtype C had a higher genetic barrier for the G140C and G140S mutations, highlighting that the Q148H/K/R DTG resistance pathway was selected less in subtype C. Docking analysis of the DTG showed that the PR- and RT-associated HIVDRM did not affect the structure of the HIV-1 IN, supporting the use of DTG as a salvage therapy for patients with resistance to drugs targeting these enzymes.

The absence of major INSTI DRMs among INSTI-naïve patients in our study was consistent with other studies from Africa [58,59,60,61,62,63,64], Asia [65,66,67], and Europe [68,69,70], showing no or highly infrequent major INSTI mutations among INSTI-naïve patients. Our finding was not unexpected, and was in line with studies from other settings based on samples obtained before the rollout of DTG [71,72,73]. However, following the wide scale-up of DTG, an increase in DTG resistance has been reported, especially in persons receiving DTG monotherapy [15,19,23,24,25,26,27,28]. Hitherto, the prevalence of transmitted resistance to DTG resistance has been low [20,21,22,23]. Similarly, in Ethiopia, after implementing the test-and-treat strategy, an increased number of patients will be on a DTG-based regimen. Thus, the emergence of INSTI resistance is expected, especially in settings with low access to viral load monitoring, delaying the identification of patients with treatment failure and increasing the risk of HIV drug resistance [74]. 

When present alone, accessory mutations have a minimal effect on INSTI susceptibility, but may serve to augment resistance and/or restore the fitness of viral mutants with major resistance mutations [5,30]. INSTI accessory mutations were detected in 20 (4.4%) of our specimens, and were equally distributed in both ART-naive and ART-experienced patients. Similar to our findings, different studies [67,72,73,75] revealed that NOPs were common among INSTI-naive patients. However, the prevalence differed with HIV-1 subtypes or circulating recombinant forms. 

E157Q was the most common nonpolymorphic accessory mutation detected in our analysis. It is a natural polymorphism present in 1–10% of untreated individuals, depending on the subtype. It has no effect on the susceptibility of INSTI. However, it may act as a compensatory substitution for R263K-induced resistance to DTG [76]. Q95K was among the other nonpolymorphic accessory INSTI resistance detected in our study, and it had little, if any, effect on drug susceptibility to INSTI; however, in the presence of a N155H mutation, it increased INSTI resistance and improved the impaired replication of the virus [77]. 

L74M/I (2.9%) and M50I (18.8%) were the other polymorphic mutations detected in our study. L74M/I has been reported at levels between 0.5–20% in the untreated population, with a high prevalence in subtypes A, G, and A/G recombinants. It does not decrease INSTI susceptibility alone, but it can contribute to a high-level resistance when occurring with major INSTI-resistance mutations, mainly the Q148H/K/R mutation [24,58,78,79]. Studies in South Africa, Brazil and Europe have also confirmed a low frequency of L74M in INSTI-naïve patients [64,68,80]. M50I can be found in 10–25% of INSTI-naïve patients [81]. M50I alone does not negatively impact integrase strand-transfer activity and HIV replication capacity, but in combination with R263K, it increased resistance to DTG by 15.6-fold [81]. 

The other nonpolymorphic and polymorphic accessory mutations detected were G163R and T97A, which can contribute to a high-level resistance when occurring with Y143 and N155H major INSTI-resistance mutations [30]. 

In this study, we characterized the distribution of amino acid variants among the 453 HIV-1 subtype C IN sequences from INSTI-naïve individuals. Our results revealed that 64.9% of HIV-1 IN amino acid positions were conserved (<1.0% variability). The conserved position in the NTD, CCD, and CTD were 60%, 66.0%, and 65.8%, respectively. This was comparable to the study by Rhee et al. (2008) that showed 70% (202/288) of IN amino acid positions of the 1500 sequences obtained from INSTI-naïve (ART-naive or ART-experienced) individuals with different subtypes (<1.0% variability) [5]. Similarly, Hackett et al. (2008) also showed that 65% (187/288) of amino acid positions were conserved after analyzing 1304 HIV-1 sequences from groups M, N, and O IN sequences [82]. 

In general, our results showed that the majority of amino acids involved in key functions of the enzyme, including the zinc-binding HHCC motif, the multimerization of IN subunits, and the binding with the human cellular factor LEDGF/p75 in the catalytic core domain, the catalytic triad DDE [6,22] was highly conserved. The high conservation might have been due to the absence of INSTI pressure. All of our study participants were INSTI-naïve, and INSTI was not used in Ethiopia during our sample collection. However, a highly polymorphic residue in the NTD, CCD, and CTD regions, which might have affected the IN-protein function and interfered with the INSTI binding, were also observed [22,30]. Further long-term treatment follow-up studies are needed to assess the potential impact of NOPs on the evolution of INSTI resistance and viral fitness under the pressure of INSTIs.

It was also interesting to note that 20.5% (93/453) of our study participants were found to harbor a major HIVDR mutation (transmitted and acquired HIVDR) for NRTI, NNRTI, and/or PI in their corresponding PR/RT gene. However, DRM directed toward sites other than IN did not have a significant effect on INSTI susceptibility. In line with our findings, different studies have shown that previous NRTIs mutations appeared to have no impact on the risk of virological failure in patients switched to DTG with NNRTIs [83,84,85,86]. However, this was in contrast to other studies that showed previous exposure to NNRTI, PI, and/or NNRTI induced mutations or increase polymorphisms in the IN gene, highlighting the functional cooperation between viral IN and RT, and/or a potential coevolution of some of their mutations [9,87]. For instance, a study by Ceccherini et al. (2009, 2010) showed a higher frequency of I84V, M154I, and V165I among ART-treated subtype B patients compared to ART-naïve patients, implying that nonsuppressive ART treatment based on other antiretroviral drug classes (NRTI and/or NNRTI) might induce IN polymorphisms [6,9]. 

However, in our study, no significant difference was found in I84V and M154I prevalence between the ART-naïve and ART-experienced patients (22.6% and 0.5% of I84V and M154I among ART-naïve, and 1.15% and 12.6% among ART-experienced patients, respectively (*p* = 0.5 and *p* = 0.4)), while an increased prevalence of V165I was observed among ART-experienced groups (5.45% of V165I and12.64% between the ART-naïve and ART-experienced groups, respectively (*p* = 0.03)). Furthermore, our comparison of the HIVDR and no-HIVDR groups showed no differences (17.4%, 0.4%, and 7.3% of I84V, M154I, and V165I for the no-HIVDR group; and 22.6%, 1.1%, and 6.5% for the HIVDR group; *p* = 0.4, *p* = 0.5, and *p* = 1, respectively).

The observed differences between this and previous studies might be due to the number of sequences, range of major/minor mutations, and subtypes included in the analysis. However, the lack of a major INSTI mutation among sequences with multiple mutations in the PR/RT gene and the high conservation of amino acids involved in key functions of the IN enzyme did not support the impact of previous ART treatment on INSTI susceptibility. 

Our docking analysis further supported our results, and showed no differences between the HIVDR and no-HIVDR groups. In both groups, DTG was successfully docked at a similar position to the original DTG ligand with the best docking score of −6.5 kcal/mol.

The genetic barrier, which is a crucial factor in the development of drug resistance, is defined by a cumulative number of resistance-associated mutations (RAMs) required for the virus to escape drug-selective pressure [53]. It is an important factor that contributes to the development of drug resistance. The variability at the nucleotide level in the IN among the different subtypes could influence the genetic barrier of INSTI drugs. In this study, we explored how the variability between subtypes C and B could affect DTG resistance.

Overall, our analysis of the codon distribution of the selected amino acid position of HIV-1 subtype C and subtype B revealed a similar genetic barrier for the development of DTG resistance between subtype C and B, except at codon position 140, where subtype C had a higher genetic barrier to develop the G140C and G140S mutations compared to subtype B, highlighting a higher genetic barrier for the Q148H/R/K resistance pathway in subtype C. The G140S mutation has been shown to rescue the catalytic defect due to the Q148H mutation, enabling the recovery of viral fitness [88]. A similar high genetic barrier to acquire mutations G140S or G140C has also been described in CRF02_AG compared with subtype B [53,89]. 

This study was comprehensive, and included both treatment-naïve and treatment-experienced (first- and second-line regimens) patients, and will be a benchmark for INSTI DRM monitoring in Ethiopia. However, our analysis was based on the Sanger dideoxy sequencing method, which does not detect drug-resistance minority variants below 20% of the virus population, and might have underestimated the prevalence of INSTI DRMs among our study participants [90].

## 5. Conclusions

Our results showed no major clinically relevant INSTI-associated mutations among INSTI-naïve patients regardless of exposure to other antiretroviral agents, supporting the implementation of the wide scale-up of DTG-based regimes in Ethiopia. However, the detection of polymorphisms contributing to INSTI resistance and the expected increased use of DTG-based regimens in Ethiopia warrant the need for continuous surveillance of INSTI resistance. The genetic barrier analysis showed that subtype C had a high genetic barrier to acquiring the G140C and G140S mutations, highlighting that the Q148H/K/R mutation DTG resistance pathway was selected less in subtype C. Moreover, the docking analysis of the dolutegravir showed that protease- and reverse-transcriptase-associated HIVDRMs did not affect the native structure of the HIV-1 integrase.

## Figures and Tables

**Table 1:** Prevalence of integrase accessory mutations detected and their ART status.

No.	Sequence ID	ART Regimen	Age	Gender	CD4+ T-Cell Count (Cells/mm^3^)	Viral Load (Copies/mL)	INSTI Accessory Mutation
1	ETH-0186	Naive	40	M	359	62,118	E157Q
2	ETH-0232	Naive	42	M	83	--	G163R
3	ETH-0343	Naive	40	M	42	26,531	E157Q
4	ETH-0358	Naive	35	M	69	418,611	G149A, E157Q
5	ETH-0366	Naive	27	F	175	62,517	E157Q
6	ETH-0380	Naive	34	M	16	397,306	E157Q
7	ETH-0396	Naive	25	F	341	47,435	G163R
8	ETH-0410	Naive	38	F	538	11,458	E157Q
9	ETH-0493	Naive	45	M	164	7130	Q95K
10	ETH-0508	Naive	39	M	150	--	E157Q
11	ETH-0545	Naive	30	F	--	--	E157Q
12	ETH-0609	Naive	28	F	895	2002	T97A
13	ETH-0622	Naive	21	F	236	155,331	T97A
14	ETH-0631	Naive	35	F	50	295,532	E157Q
15	ETH-0695	Naive	35	F	--	6465	E157Q
16	ETH-0750	TDF+3TC+EFV	46	M	--	18,681	Q95K
17	ETH-0815	TDF+3TC+EFV	50	M	384	--	Q95K
18	ETH-0839	ABC+3TC+ATV/r	40	M	432	1432	G163K
19	ETH-0843	AZT+3TC+LPV/r	39	M	733	4752	G140E
20	ETH-0879	TDF+3TC+ATV/r	50	F	655	2667	E157Q

Abbreviations: age, in years; F, female, M, male; ART, antiretroviral therapy; INSTI, integrase strand-transfer inhibitor; 3TC, lamiduvine; TDF, tenofovir, AZT, zidovudine; EFV, efavirenz; LPV/r, lopinavir/ritonavir; ATV/r, atazanavir/ritonavir; Naïve, ART-naive; ”--”, missing data. CD4+ T in cells/mm^3^; HIV RNA in copies/mL.

**Table 2:** Analysis of genetic barrier based on the minimum number of transitions and transversions required to obtain mutation resistance to DTG.

Codon Position	Substitution	Subtype C, n (%) ^a^	Subtype B, n (%) ^b^	Wild-Type Codon	Mutant Codon	Minimal Score ^c^
66	T66A	439 (96.91)	1829 (97.08)	ACA	GTC, GCC/A/G	1
		3 (0.66)	7 (0.37)	ACG		1
		3 (0.66)	6 (0.32)	ACT		2
		8 (1.77)	42 (2.23)	ACC		1
	T66K	439 (96.91)	1829 (97.08)	ACA	AAA/G	2.5
		3 (0.66)	7 (0.37)	ACG		2.5
		3 (0.66)	6 (0.32)	ACT		5
		8 (1.77)	42 (2.23)	ACC		5
	T66I	439 (96.91)	1829 (97.08)	ACA	ATT/C/A	1
		3 (0.66)	7 (0.37)	ACG		3.5
		3 (0.66)	6 (0.32)	ACT		1
		8 (1.77)	42 (2.23)	ACC		1
92	E92Q	441 (97.35)	446 (23.67)	GAA	CAA/G	2.5
		12 (2,65%)	1438 (76.33)	GAG		2.5
118	G118R	394 (86.98)	1750 (92.89)	GGC	CGT/C/A/G, AGA/G	2.5
		19 (4.19)	32 (1.7)	GGA		1
		1 (0.22)	5 (0.27)	GGG		1
		39 (8.61)	91 (4.83)	GGT		2.5
138	E138A	440 (93.16)	1831 (97.19)	GAA	GTC, GCC/A/G	2.5
		13 (2.87)	39 (2.07)	GAG		2.5
	E138K	440 (93.16)	1831 (97.19)	GAA	GTC, GCC/A/G	1
		13 (2.87)	39 (2.07)	GAG		1
	E138T	440 (93.16)	1831 (97.19)	GAA	ACT/C/A/G	3.5
		13 (2.87)	39 (2.07)	GAG		3.5
140	G140A	243 (53.64)	18 (0.96)	GGG	GTC, GCC/A/G	2.5
		208 (45.92)	58 (3.08)	GGA		2.5
		1 (0.22)	201 (10.67)	GGT		3.5
		1 (0.22)	1607 (85.30)	GGC		2.5
	G140S	243 (53.64)	18 (0.96)	GGG	TCT/C/A/G, AGT/C	3.5
		208 (45.92)	58 (3.08)	GGA		3.5
		1 (0.22)	201 (10.67)	GGT		1
		1 (0.22)	1607 (85.30)	GGC		1
	G140C	243 (53.64)	18 (0.96)	GGG	TGT, TTC	5
		208 (45.92)	58 (3.08)	GGA		5
		1 (0.22)	201 (10.67)	GGT		2.5
		1 (0.22)	1607 (85.30)	GGC		3.5
143	Y143C	436 (96.25)	1877 (99.63)	TAC	TGT, TTC	2.5
		17 (3.75)	7 (0.37)	TAT		1
	Y143H	436 (96.25)	1877 (99.63)	TAC	CAT/C	1
		17 (3.75)	7 (0.37)	TAT		1
	Y143R	436 (96.25)	1877 (99.63)	TAC	CGT/C/A/G. AGA/G	3.5
		17 (3.75)	7 (0.37)	TAT		2
147	S147G	403 (88.96)	1828 (97.03)	AGT	GGT/C/A/G	1
		50 (11.04)	56 (2.97)	AGC		1
148	Q148H	71 (15.67)	1828 (97.03)	CAA	CAT/C	2.5
		382 (84.33)	56 (2.97)	CAG		2.5
	Q148K	71 (15.67)	1828 (97.03)	CAA	AAA/G	2.5
		382 (84.33)	56 (2.97)	CAG		2.5
	Q148R	71 (15.67)	1828 (97.03)	CAA	CGT/C/A/G, AGA/G	1
		382 (84.33)	56 (2.97)	CAG		1
155	N155H	427 (94.26)	1849 (98.14)	AAT	CAT/C	2.5
		26 (5.74)	35 (1.86)	AAC		2.5
263	R263K	62 (13.69)	1833 (97.29)	AGA	AAA/G	1
		389 (85.87)	4 (0.21)	AGG		1

^a^ Subtype C: Ethiopian sequence used in the analysis (*n* = 453). ^b^ Subtype B: global subtype B sequence deposited before 2007 (before INSTI was used) retrieved from the Los Alamos database (*n* = 1884). ^c^ Minimal score calculated by the sum of number of transversions and transitions for each, with transitions scored as 1 and transversions scored as 2.5.

## Data Availability

All the sequences from this study were deposited in the GenBank with accession numbers OM302554–OM303013.

## Supplementary Materials

The following supporting information can be downloaded at: https://www.mdpi.com/article/10.3390/v14040729/s1, Table S1: Type of HIV-drug-resistance mutations detected in the HIVDR group (patients with one or more major HIVDR mutation to NRTI, NNRTI, and/or PI) (*n* = 93), Table S2: Modifications of the 6u8q.pdb HIV-1 integrase structure according to the alignment of both the ADR and PDR sequences.
