# Supplementary material for: Pre-Treatment Integrase Inhibitor Resistance and Natural Polymorphisms among HIV-1 Subtype C Infected Patients in Ethiopia
Source: Viruses. 2022 Mar 30;14(4):729. doi: 10.3390/v14040729 (PMC9029575; doi:10.3390/v14040729)
Supplement: Supplementary file 1 [file viruses-14-00729-s001.zip › Table S1.pdf]

Table S1: Type of HIV drug resistance mutations detected among HIVDR-group (patients with one or more major HIVDR mutation to NRTI, NNRTI and /or PI), (n=93)

Sequence Name	Age	Gender	Regimen	CD4 (cells/ mm3)	VL (copies/ml)	PI Major	NRTI major HIVDRMs	NNRTI major HIVDRMs	INSTI major HIVDRMs	INSTI Accessory
ETH-0888	38	F	AZT+3TC+LPV/r	177	2,867	None	None	V106M, E138EA, G190A	None	None
ETH-0887	25	F	AZT+3TC+LPV/r	244	1,263	None	D67N, K70R, M184V, T215V, K219Q	K101E, G190A	None	None
ETH-0882	40	M	AZT+3TC+LPV/r	25	12,192	None	A62V, K65R, Y115F, M184V	K103N, V106M	None	None
ETH-0880	39	M	TDF+3TC+ATV/r	--	5,167	None	M41L, E44ED, L74LI, M184V, L210W, T215Y	K103N, V108VI, P225H	None	None
ETH-0879	30	F	TDF+3TC+ATV/r	53	2,667	None	D67N, K70R, M184V, T215Y, K219E	L100I, K103N	None	E157Q
ETH-0877	50	M	ABC+3TC+ATV/r	53	66,013	None	D67N, K70R, M184V, T215Y, K219E	L100I, K103N	None	None
ETH-0868	30	M	AZT+3TC+LPV/r	99	6,465	M46I, I54V, V82A	A62V, T69S_SA, M184V, T215Y	A98G, Y181C, H221Y	None	None
ETH-0866	40	F	AZT+3TC+LPV/r	43	1,447	V82A	K70G, Y115F, M184V	Y181C, H221Y	None	None
ETH-0858	24	F	ABC+3TC+ATV/r	67	128,051	None	None	Y181I	None	None
ETH-0857	37	F	TDF+3TC+LPV/r	605	5,789	M46I, I54V, N88S	M41L, E44D, D67N, M184V, L210W, T215Y	None	None	None
ETH-0850	30	M	ABC+3TC+ATV/r	178	568,757	V82A, L90M	K65R, D67N, Y115F, M184V, K219E	V106I, Y188L, H221Y	None	None
ETH-0849	46	F	TDF+3TC+LPV/r	53	37,655	None	None	K103N	None	None
ETH-0848	33	F	TDF+3TC+LPV/r	44	1,035	None	None	K103N	None	None
ETH-0847	50	M	TDF+3TC+LPV/r	23	166,432	None	None	V108VI, Y181C, H221Y	None	None
ETH-0840	30	F	ABC+3TC+LPV/r	49	1,319	None	K65R, M184V, K219E	Y181C, G190A	None	None
ETH-0832	41	F	TDF+3TC+ATV/r	78	7,681	None	M184MV	K101AE, G190A	None	None
ETH-0831	31	M	TDF+3TC+LPV/r	38	352,9	None	E44D	K103N, K238T	None	None
ETH-0829	22	M	AZT+3TC+ATV/r	21	269,678	M46I, I50IL, I54IV, V82A	K70E, Y115F, M184V	A98G, K101E, Y181C, G190A	None	None
ETH-0826	48	F	TDF+3TC+EFV	--	2,942	None	None	K101KHNQ, Y188L	None	None
ETH-0824	19	M	TDF+3TC+EFV	--	30,090	None	M41L, E44ED, D67G, T69D, K70R, M184V, L210W, T215F,K219Q	A98G, V108I, Y181C	None	None
ETH-0822	18	F	D4T+ 3TC+ NVP	--	65,918	None	V75VM	K103N, P225PH	None	None
ETH-0816	34	M	TDF+3TC+EFV	--	19,894	None	K70KE, M184V	K103N, V108VI, H221Y	None	None
ETH-0799	27	F	AZT+ 3TC+ NVP	--	82,927	None	K65R, L74V, Y115F	E138K, G190E, H221Y	None	None
ETH-0798	30	F	D4T+ 3TC+ NVP	--	98,531	None	None	V179D, Y188C	None	None

Table S1: Type of HIV drug resistance mutations detected among HIVDR-group (patients with one or more major HIVDR mutation to NRTI, NNRTI and /or PI), (n=93)

ETH-0797	32	M	AZT+3TC+EFV	--	3,365	None	K70KE, M184V	K101P, K103N	None	None
ETH-0796	37	M	D4T 3TC NVP	--	91,965	None	None	K103N	None	None
ETH-0795	47	M	D4T+ 3TC+ NVP	--	39,162	None	A62V, K65R, Y115F, M184V	V106M, V179D	None	None
ETH-0792	36	F	D4T+ 3TC+ NVP	--	1,305	None	D67N, K70R, M184V, K219E	Y188L	None	None
ETH-0784	18	M	D4T+ 3TC+ NVP	--	239,35	M46MI	K65R, K70R	E138A, G190EK, H221Y	None	None
ETH-0783	28	F	D4T+ 3TC+ NVP	--	540,736	None	K65R, D67G, M184V	K101KE, Y181C, G190T	None	None
ETH-0780	23	M	D4T+ 3TC+ NVP	--	80,116	None	K70KQ, L74LI, M184I	K103N, H221Y, P225H	None	None
ETH-0779	57	M	D4T+ 3TC+ NVP	--	4,839	None	None	K103N	None	None
ETH-0777	18	M	D4T+ 3TC+ NVP	--	88,848	None	M184V, T215Y	K101E, G190A	None	None
ETH-0766	37	M	TDF+3TC+EFV	--	5,958	None	None	K103KN	None	None
ETH-0765	30	F	AZT+3TC+NVP	--	17,212	None	A62V, K65R, M184V	K103N, V106M, V179T	None	None
ETH-0764	30	F	TDF+3TC+EFV	--	15,926	None	None	Y188L	None	None
ETH-0757	32	M	TDF+3TC+EFV	--	515,429	None	K70KQ	K103KN, V106VM	None	None
ETH-0751	44	F	TDF+3TC+EFV	--	3,805	None	M184V	K101E, G190A	None	None
ETH-0750	46	M	TDF+3TC+EFV	--	18,681	None	A62AV, K65R	K101EQ, V179IT, Y181YC, G190S	None	Q95K
ETH-0749	26	M	TDF+3TC+EFV	--	77,025	None	A62AV, K65R, M184V	L100I, K103N	None	None
ETH-0748	35	F	AZT+3TC+EFV	--	10,414	None	D67N	K103N	None	None
ETH-0744	35	F	AZT+3TC+EFV	--	5,717	None	None	K103N	None	None
ETH-0741	39	M	AZT+3TC+NVP	--	3,944	None	M184V	K103N, P225H	None	None
ETH-0727	38	F	AZT+3TC+NVP	--	13,365	None	K70E, M184V, L210LW	A98G, L100I, K103N	None	None
ETH-0725	35	F	AZT+3TC+NVP	--	6,926	None	D67DN, M184V	V106M, Y181C, H221Y	None	None
ETH-0720	30	F	Naïve	170	--	None	None	K103N, V106M, G190A	None	None
ETH-0712	30	M	Naïve	450	829,757	None	E44D	None	None	None
ETH-0705	27	F	Naïve	210	1,65	None	L210W	None	None	None
ETH-0698	45	M	Naïve	1242	--	None	E44D	E138EA	None	None
ETH-0688	28	F	Naïve	291	124,567	None	D67N, K70E,M184V	K101HN, V106M, G190A,F227FL	None	None
ETH-0683	30	F	Naïve	110	2,33	None	None	V106M	None	None

Table S1: Type of HIV drug resistance mutations detected among HIVDR-group (patients with one or more major HIVDR mutation to NRTI, NNRTI and /or PI), (n=93)

ETH-0650	35	F	Naïve	827	22,062	None	None	F227L	None	None
ETH-0637	45	F	Naïve	115	276,867	None	D67DANT, K70KR, M184V, T215F	A98AG, K101HQ, Y181C	None	None
ETH-0620	30	F	Naïve	--	59,332	None	K70KR	K103KN, Y181C	None	None
ETH-0617	31	F	Naïve	--	2,879	None	None	A98G	None	None
ETH-0603	26	F	Naïve	560	325,96	None	K70KE, M184MIV	Y181YC, G190A	None	None
ETH-0591	49	M	Naïve	--	45,606	None	None	E138A	None	None
ETH-0580	30	F	Naïve	241	21,76	None	None	K103N	None	None
ETH-0577	43	M	Naïve	221	177,072	None	None	K103N	None	None
ETH-0569	34	F	Naïve	662	--	None	K65KR, M184MV	K103N, V108VI, G190GA	None	None
ETH-0556	37	F	Naïve	557	130,642	None	None	K101E	None	None
ETH-0547	33	F	Naïve	--	1,137	None	None	K103T	None	None
ETH-0545	37	F	Naïve	778	86,43	None	None	E138A	None	E157EQ
ETH-0539	25	F	Naïve	--	66,282	None	None	K103N	None	None
ETH-0505	30	F	Naïve	--	--	None	None	K103KN	None	None
ETH-0498	42	M	Naïve	41	--	None	K70R, M184V, K219Q	K101E, Y181C	None	None
ETH-0479	45	F	Naïve	--	200,475	None	A62AV, K65KR, M184MV	None	None	None
ETH-0478	28	F	Naïve	305	73,246	None	K70E, M184V	A98G, K103N, Y181C, G190A	None	None
ETH-0466	45	F	Naïve	115	14,263	None	None	E138A	None	None
ETH-0449	35	M	Naïve	307	23,801	None	K219N	V108VI, Y181C	None	None
ETH-0412	21	M	Naïve	158	785,371	None	None	K103T, V106VA	None	None
ETH-0411	38	M	Naïve	--	10,843	None	A62AV	None	None	None
ETH-0404	36	F	Naïve	--	134,072	None	None	G190GA	None	None
ETH-0383	30	F	Naïve	128	372,286	None	K70KR, M184MV	V108VI, V179VD, Y181YC, H221HY	None	None
ETH-0376	35	F	Naïve	118	36,441	None	None	K103N	None	None
ETH-0342	25	M	Naïve	--	81,977	None	L210W	None	None	None
ETH-0339	29	M	Naïve	114	26,928	None	None	K103N	None	None

Table S1: Type of HIV drug resistance mutations detected among HIVDR-group (patients with one or more major HIVDR mutation to NRTI, NNRTI and /or PI), (n=93)

ETH-0324	23	F	Naïve	--	114,673	None	None	V179VD	None	None
ETH-0302	50	M	Naïve	16	2,174	None	None	K103N	None	None
ETH-0267	30	M	Naïve	25	471,458	None	None	K103N	None	None
ETH-0263	23	F	Naïve	514	218,073	None	None	E138A	None	None
ETH-0252	37	F	Naïve	29	727,997	None	None	K103N, E138A	None	None
ETH-0229	40	F	Naïve	430	883,029	None	None	A98G	None	None
ETH-0209	35	F	Naïve	--	14,263	None	None	K103KN, V106VM, G190GA	None	None
ETH-0203	40	F	Naïve	1147	27,275	None	L210W	None	None	None
ETH-0194	27	F	Naïve	423	57,182	M46ML	None	None	None	None
ETH-0156	37	M	AZT+3TC+ATV/r	51	197,002	None	K65R, Y115F, M184V	K103N, Y181C	None	None
ETH-0154	42	F	TDF+3TC+ATV/r	165	36,29	M46I, I54V, V82A	E44D, M184V, L210W, T215Y	V108I, H221Y	None	None
ETH-0153	50	F	TDF+3TC+LPV/r	24	321,361	None	D67DG, T69TADN	L100LI, K103N	None	None
ETH-0151	38	F	TDF+3TC+LPV/r	83	5,226	None	None	A98G, V179D	None	None
ETH-0121	32	M	D4T+ 3TC+ NVP	--	--	None	None	K103N, G190A	None	None
ETH-0070	24	F	Naïve	--	779,973	None	None	E138A	None	None
ETH-0036	41	M	Naïve	--	767,986	None	None	E138A	None	None

Age: in years; Gender: F, Female; M, Male; CD4+ T in cells/mm3; HIV RNA in copies/ml; PI, Protease inhibitors; NRTI; nucleoside reverse transcriptase inhibitor; NNRTI ; non-nucleoside reverse transcriptase inhibitors, INSTI, Integrase strand transfer inhibitors; HIVDRMs; HIV drug resistance mutation, 3TC, lamiduvine; D4T, stavudine; TDF, tenofovir, ZDV/ AZT, zidovudine; EFV, efavirenz; NVP, nevirapine; ABC, Abacavir; LPV/r, Lopinavir/ritonavir; ATV/r, Atazanavir/ritonavir; Naïve, ART-naive; "--": for missing data; "None", not detected
