# Supplementary material for: Pre-Treatment Integrase Inhibitor Resistance and Natural Polymorphisms among HIV-1 Subtype C Infected Patients in Ethiopia
Source: Viruses. 2022 Mar 30;14(4):729. doi: 10.3390/v14040729 (PMC9029575; doi:10.3390/v14040729)
Supplement: Supplementary file 1 [file viruses-14-00729-s001.zip › Table S2.pdf]

Table S2. **Modifications of 6u8q.pdb HIV-1 integrase structure according to the alignment of both ADR and PDR sequences**

	Position*	Substitution
Valine	31	Isoleuine
Valine	72	Isoleuine
Isoleuine	101	Leucine
Threonine	112	Valine
Threonine	113	Isoleuine
Glyine	123	Serine
Threonine	124	Alanine
Threonine	125	Alanine
Lysine	136	Glutamic acid
Isoleuine	151	Valine
Valine	201	Isoleuine
Threonine	218	Leucine

* Position according to amino acid sequence of chain A of 6U8Q HIV-1 integrase.
